# Melanosis Peritonei Associated With Adenocarcinoma of the Sigmoid Colon

**DOI:** 10.7759/cureus.16693

**Published:** 2021-07-28

**Authors:** Mohammed Barghash, Suad Nassif, Yazan Alkurdi, Amany Said, Moustafa Mansour

**Affiliations:** 1 General Surgery, North Manchester General Hospital, Manchester, GBR; 2 Histopathology, Northern Care Alliance Hospitals, Manchester, GBR

**Keywords:** melanosis peritonei, colorectal cancer, inguinal hernia repair, hernial sac, peritoneal melanosis

## Abstract

Melanosis peritonei is an unusual and uncommon condition characterized by pigment deposition in the peritoneum. It is usually associated with other conditions. During a right inguinal hernia open repair for an 86-year-old gentleman, widespread black spots were observed on the hernial sac. After appropriate histopathological and immunohistochemical studies, the diagnosis of melanosis peritonei was established. The past medical history of the patient included an endoscopic excision of a sigmoid polyp which was found to be an adenocarcinoma with <1mm clearance margin on histopathology. Being recognized as an extremely rare condition, melanosis peritonei should be differentiated from other conditions including metastatic melanoma.

## Introduction

Melanosis Peritonei is an unusual condition characterized by focal or diffuse pigment deposition in the peritoneum [1]. Previously reported cases have been associated with other conditions including mature ovarian cystic teratomas [1-6], an enteric duplication cyst [7,8], an ovarian serous cystadenoma [9], a peritoneal cyst [10], and gastric triplication [11]. Only two reported cases to date were associated with adenocarcinoma of the colon [12,13]. In this report, we present a case of peritoneal melanosis in an adult male previously diagnosed with adenocarcinoma of the sigmoid colon.

## Case presentation

An 86-year-old gentleman was referred from his general practitioner (GP) with a computed tomography (CT) proven large right inguinal hernia containing small bowel loops. His past medical history included hypertension and chronic obstructive pulmonary disease (COPD) for which he was on inhalers. Following the appropriate clinical examination and proper discussion, he was added to the waiting list for an elective open mesh repair.

**Figure 1:**
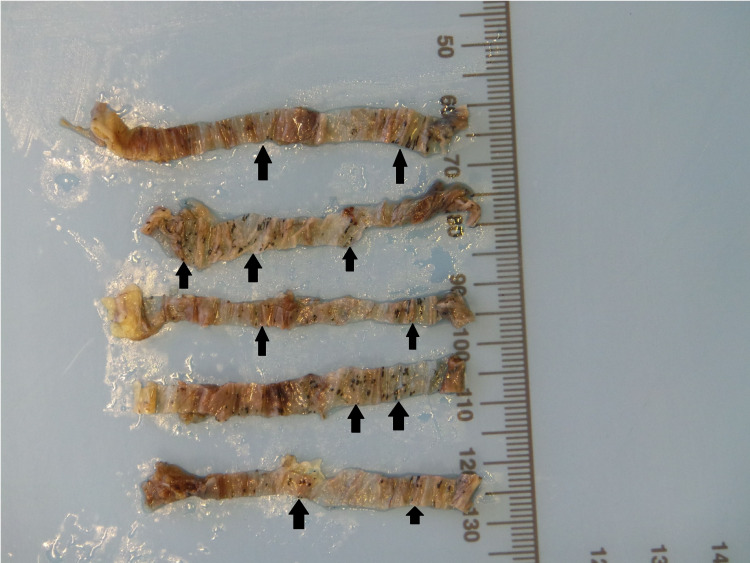
Gross photo for the hernia sac after slicing showing mottled black pigmentation of the thin sac wall (highlighted by the multiple black arrows)

**Figure 2:**
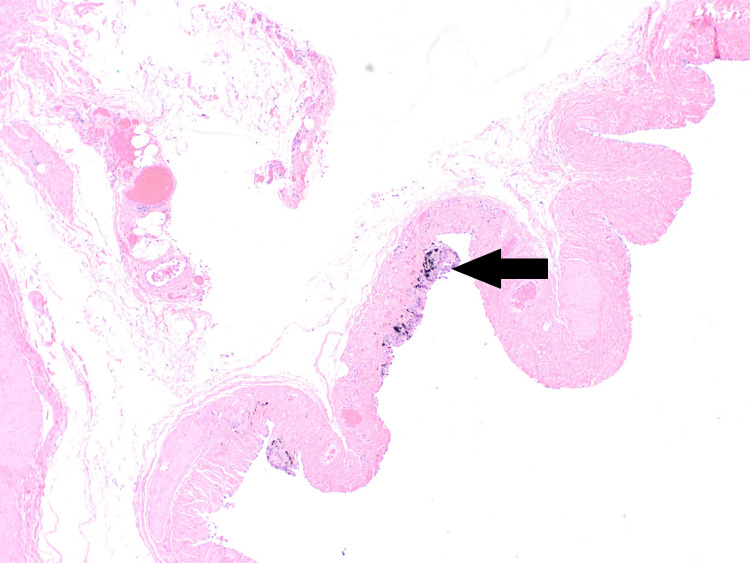
Low power view of H&E stained section from the peritoneal sac showing patchy dark black pigmentation in the sub mesothelial tissue of the sac (Black arrow) H&E : Hematoxylin and Eosin

**Figure 3:**
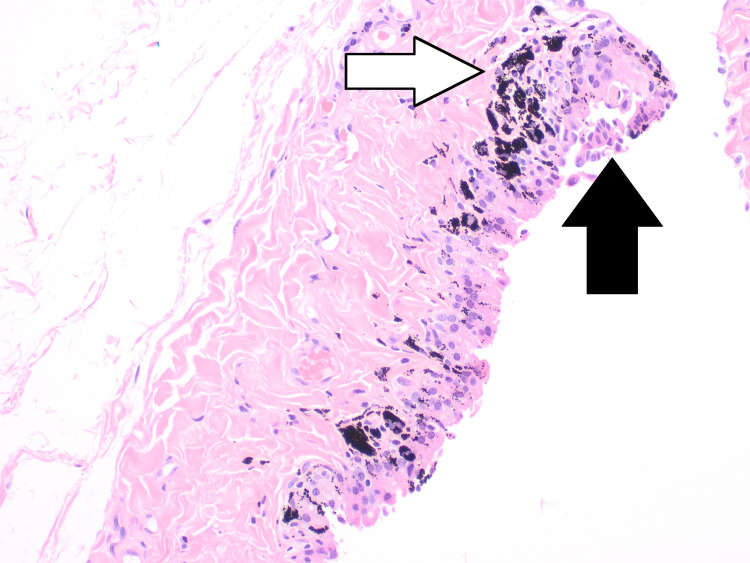
High power view of the sac wall (H&E) showing granular black pigment in the sub mesothelial tissue (White Arrow). The overlying mesothelial lining appears irritated and mildly hyperplastic (Black arrow)

**Figure 4:**
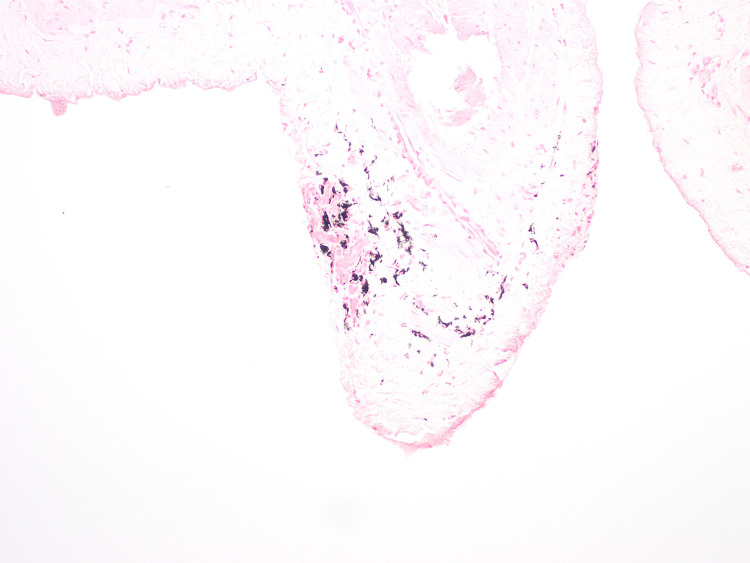
The black pigment is negative for Masson Fontana stain

**Figure 5:**
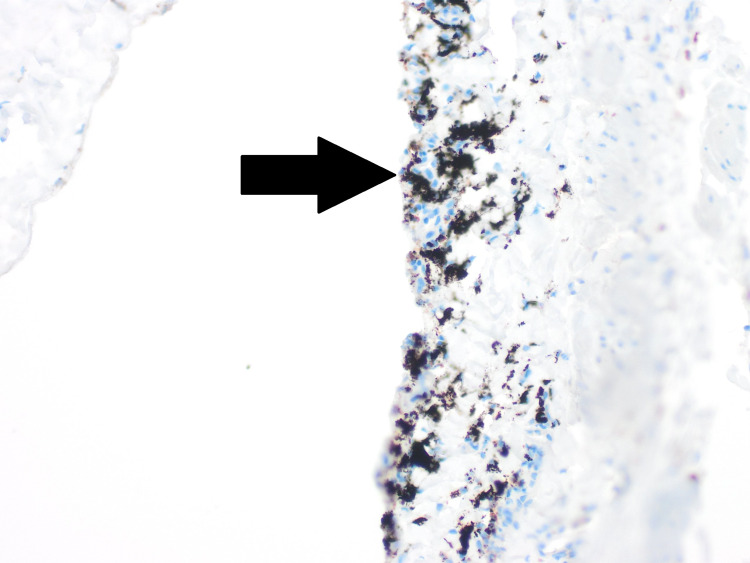
CD68 highlights macrophages engulfing the pigment (Black arrow).

During the hernia repair, widespread black spots were noted on the hernial sac (Figure 1). The hernial sac was transfixed, excised, and sent for histopathological assessment. Microscopic examination reported patchy dark black pigmentation in the sub-mesothelial tissue of the hernial sac with no evidence of dysplasia or malignancy (Figure 2). Upon viewing with high-power microscopic examination (Hematoxylin and Eosin x 20), a granular black pigment in the sub-mesothelial tissue was observed. The overlying mesothelial lining appeared irritated and mildly hyperplastic (Figure 3). In addition, Melanin pigment was not detected with Mason Fontana stain (Figure 4). Immunohistochemical studies using CD68 highlighted several macrophages containing black pigment (Figure 5). Based on these results, the diagnosis of melanosis peritonei was concluded.

It was noted that the patient was being followed up by the colorectal team because of a 25 mm distal sigmoid polyp which was endoscopically resected four months prior to his hernia procedure. Histopathological assessment of the polyp reported a moderately differentiated adenocarcinoma with a <1mm clearance margin. A computed tomography (CT) scan of his chest, abdomen, and pelvis was performed as part of the staging of his sigmoid cancerous polyp and this showed no evidence of lung, liver, or bony metastases.

The colorectal multidisciplinary team earlier concluded that there was more than a 20% risk of having residual disease and that would favour the need for undertaking a sigmoid colectomy or high anterior resection depending on his fitness.

Due to his age, comorbidities, and his baseline fitness, the patient eventually decided not to proceed with surgical resection. Therefore, a follow up was arranged in the form of regular carcinoembryonic antigen (CEA) levels, lower gastrointestinal (GI) endoscopy, and a repeat CT scan in six months.

## Discussion

To our knowledge, only 17 cases of melanosis peritonei have been reported in the English literature to date [1-17]. It is almost exclusively associated with other conditions: six with ovarian cystic teratomas [1-6], two with enteric duplication [7,8], one with gastric triplication [11], one with peritoneal cyst [10], one with serous cystadenoma of the ovary [9], one with mucinous cystadenoma of the ovary and adenocarcinoma in the colon [12], one with adenocarcinoma of the rectum [13], one with Peutz-Jeghers syndrome [14], and three with metastatic melanoma [15-17].

Peritoneal melanosis commonly affects females from 18 to 28 years of age, with only one male case reported [13]. It also has been reported in the elderly but always in association with malignancy. The affected organs are variable and include the peritoneum, ovaries, omentum, and appendix [1].

The pathogenesis of this condition is still unclear [13]. One theory, which was linked to ovarian teratoma, stated that the melanin pigment was produced inside the ovarian teratoma and spilled into the peritoneum following rupture of the cystic ovarian lesion [2-4,6,10]. This theory was widely accepted but it failed to explain the peritoneal melanosis not associated with ovarian teratoma or in cases in which the ovarian teratoma did not rupture [9].

Several other theories were introduced. One theory stated that excessive migration of neural crest cells prior to the 10th week of development leads to the formation of pigmented mesothelial cells and dendritic melanocytes in the peritoneum [10]. Another theory agreed that the pigmentation still originated from neural crest cells but instead of migration, it was due to a defect in regression of neuro-enteric canal leaving residual neural crest cells [11]. Moreover, some authors speculated that the pigment was derived from hemorrhage into the teratoma containing gastric mucosa and peptic ulceration [5]. It goes without saying that peritoneal melanosis associated with malignant melanoma was proposed to be derived from the tumor cell pigment [15,16].

The main differential diagnosis of melanosis peritonei is metastatic melanoma which may present with the same picture but with a very poor prognosis [15,16]. Although they might mimic each other grossly, metastatic melanoma usually forms large masses with frank hemorrhage rather than discrete nodules. Microscopically, the neoplastic nature of the lesion can be readily identified in haematoxylin and eosin-stained sections [13]. Therefore, all cases with melanosis peritonei should have complete dermal and ophthalmologic examination to rule out metastatic spread of malignant melanoma [15].

Peritoneal endometriosis is another differential diagnosis that should be considered. It is usually dark brown in colour and can be easily diagnosed histologically by the presence of endometrial glands, stroma, and hemosiderin-laden macrophages. Intraperitoneal spillage and spread of India ink from preoperative endoscopic tattooing can also be misinterpreted as peritoneal melanosis although it is of no pathological significance [13].

## Conclusions

In this paper, we present an extremely rare condition known as melanosis peritonei, which was incidentally found during an open inguinal hernia repair. Melanosis peritonei is almost exclusively associated with other conditions, and in our case, the patient was previously diagnosed with adenocarcinoma of the sigmoid colon.

The pathogenesis of melanosis peritonei is still debatable. This could be attributed to the rarity of this condition, the limited number of studies performed, and the variable associated pathologies. Some argued that the cause could be melanin pigment disposition which was previously formed inside ovarian teratomas. Others speculated that it is linked to neural crest cells’ developmental errors.

The main differential diagnosis is metastatic melanoma, which carries a far worse prognosis. Despite their gross resemblance, they can be differentiated on microscopic examination. Other differential diagnoses include peritoneal endometriosis and ink spillage during endoscopic tattooing.

## References

[1] Jamkhandi DM, Gaikwad P, Singh D (2014). Melanosis peritonei in pregnancy: a case report and review of literature. Gynecol Surg.

[2] Afonso JF, Martin GM, Nisco FS, de Alvarez RR (1962). Melanogenic ovarian tumors. Am J Obstet Gynecol.

[3] Fukushima M, Sharpe L, Okagaki T (1984). Peritoneal melanosis secondary to a benign dermoid cyst of the ovary: a case report with ultrastructural study. Int J Gynecol Pathol.

[4] Sahin AA, Ro JY, Chen J, Ayala AG (1990). Spindle cell nodule and peptic ulcer arising in a fully developed gastric wall in a mature cystic teratoma. Arch Pathol Lab Med.

[5] Jaworski RC, Boadle R, Greg J, Cocks P (2001). Peritoneal “melanosis” associated with a ruptured ovarian dermoid cyst: report of a case with electron-probe energy dispersive X-ray analysis. Int J Gynecol Pathol.

[6] Lee D, Pontifex AH (1975). Melanosis peritonei. Am J Obstet Gynecol.

[7] Nada R, Vaiphei K, Rao KL (2000). Enteric duplication cyst associated with melanosis peritonei. Indian J Gastroenterol.

[8] Jung YC, Chen CJ, Tzeng CC (1996). Melanosis peritonei associated with enteric duplication cyst: a case report. Am J Surg Pathol.

[9] Kim NR, Suh YL, Song SY, Ahn G (2002). Peritoneal melanosis combined with serous cystadenoma of the ovary: a case report and literature review. Pathol Int.

[10] Drachenberg CB, Papadimitriou JC (1990). Melanotic peritoneal cyst. Light-microscopic and ultrastructural studies. Arch Pathol Lab Med.

[11] De la Torre Mondragón L, Carrasco Daza D, Bustamante AP, Fascinetto GV (1997). Gastric triplication and peritoneal melanosis. J Ped Surg.

[12] Kim SS, Nam JH, Kim SM, Choi YD, Lee JH (2010). Peritoneal melanosis associated with mucinous cystadenoma of the ovary and adenocarcinoma of the colon. Int J Gynecol Pathol.

[13] Chang ES, Bachul P, Szura M, Szpor J, Okoń K, Walocha JA (2015). Peritoneal “melanosis”. Pol J Pathol.

[14] Hirasawa A, Akahane T, Tsuruta T (2012). Lobular endocervical glandular hyperplasia and peritoneal pigmentation associated with Peutz-Jeghers syndrome due to a germline mutation of STK11. Ann Oncol.

[15] Lim CS, Thompson JF, McKenzie PR, McCarthy SW, Scolyer RA (2012). Peritoneal melanosis associated with metastatic melanoma involving the omentum. Pathology.

[16] Reccia I, Pisanu A, Podda M, Uccheddu A (2015). An uncommon presentation of metastatic melanoma: a case report. Medicine (Baltimore).

[17] Sim KK, Connell K, Bhandari M, Paton D (2021). Peritoneal melanosis associated with metastatic melanoma previously treated with targeted and immune checkpoint inhibitor therapy. BMJ Case Rep.

